# Blood mercury concentrations are associated with decline in liver function in an elderly population: a panel study

**DOI:** 10.1186/s12940-017-0228-2

**Published:** 2017-03-04

**Authors:** Mee-Ri Lee, Youn-Hee Lim, Bo-Eun Lee, Yun-Chul Hong

**Affiliations:** 1 Department of Preventive Medicine, Seoul National University College of Medicine, Seoul, Republic of Korea; 2 Institute of Environmental Medicine, Seoul National University Medical Research Center, Seoul, Republic of Korea; 3 Environmental Health Center, Seoul National University College of Medicine, Seoul, Republic of Korea; 4 Environmental Health Research Division, Environmental Health Research Department, National Institute of Environmental Research, Incheon, Republic of Korea

**Keywords:** Mercury, Liver function test, Aged, Mercury alcohol interaction

## Abstract

**Background:**

Mercury is a toxic heavy metal and is known to affect many diseases. However, few studies have examined the effects of mercury exposure on liver function in the general population. We examined the association between blood mercury concentrations and liver enzyme levels in the elderly.

**Methods:**

We included 560 elderly participants (60 years or older) who were recruited from 2008 to 2010 and followed up to 2014. Subjects visited a community welfare center and underwent a medical examination and measurement of mercury levels up to five times. Analyses using generalized estimating equations model were performed after adjusting for age, sex, education, overweight, alcohol consumption, smoking, regular exercise, high-density lipoproteins cholesterol, and total calorie intake. Additionally, we estimated interaction effects of alcohol consumption with mercury and mediation effect of oxidative stress in the relationship between mercury levels and liver function.

**Results:**

The geometric mean (95% confidence interval (CI)) of blood mercury concentrations was 2.81 μg/L (2.73, 2.89). Significant relationships were observed between blood mercury concentrations and the level of liver enzymes, including aspartate aminotransferase (AST), alanine aminotransferase (ALT), and gamma glutamyl transferase (GGT), after adjusting for potential confounders (*P* < 0.05). The odds ratios of having abnormal ALT levels were statistically significant in the highest mercury quartile compared to those with the lowest quartile. Particularly, regular alcohol drinkers showed greater effect estimates of mercury on the liver function than non-drinkers groups. There was no mediation effect of oxidative stress in the relationship between blood mercury concentrations and liver function.

**Conclusions:**

Our results suggest that blood mercury levels are associated with elevated liver enzymes and interact with alcohol consumption for the association in the elderly.

**Electronic supplementary material:**

The online version of this article (doi:10.1186/s12940-017-0228-2) contains supplementary material, which is available to authorized users.

## Background

Mercury is a toxic heavy metal that causes various adverse effects on health, including hypertension, cardiovascular disease, neurotoxicity and nephrotoxicity [1–3]. Mercury exists in various forms, including elemental (or metallic), inorganic and organic (e.g., methylmercury), which indicate multiple routes of exposure [4]. Mercury enters the body through the air, food and water, and is then distributed to all bodily tissues from the bloodstream within 3 days and subsequently eliminated slowly through feces and urine [5]. In the general population, most blood mercury levels are considered to be from organic methylmercury through fish consumption [6]. The half-life of total mercury is 57 ± 18 days in blood and 64 ± 22 days in hair [7]. Mercury is secreted into bile and partly reabsorbed by the liver through the portal vein in biliary-hepatic cycling. The high mobility of mercury in the body is due to the formation of a mercury-cysteine complex attached to the sulfur atoms of thiol groups (glutathione) in the bile duct and gall bladder [5, 8].

A recent review suggested that mercury cause neurologic disorders [9], cardiovascular disease [10], and asthma in children [11]. In addition to evidence of mercury effects on various organs, some animal studies have suggested that acute mercury exposure can damage the liver [12, 13]. However, the relationship between chronic mercury exposure and liver-related outcomes in humans is not well understood.

Epidemiologic studies of the association between mercury exposure and liver function have shown inconsistent results. A cross-sectional study in people who lived near Minamata, which was highly polluted by methylmercury, reported that the prevalence of liver disease was not significantly higher than other areas with less polluted level of methylmercury [14]. Poursafa et al. [15] showed that aspartate aminotransaminase (AST) and alanine aminotransaminase (ALT) did not increase significantly with increased quartiles of blood mercury levels. In contrast, Cave et al. [16] suggested a positive association between blood mercury levels and ALT in the US population. Lee et al. [17] showed an association between blood mercury levels and AST and ALT in 2008–2012 Korean national health and nutrition examination survey (KNHANES), and Seo et al. [18] reported an association between blood mercury levels and γ-glutamyl transferase (GGT) in 2010 KNHANES. All of these previous studies used a cross-sectional design based on a single measurement of blood mercury and liver enzyme, which does not capture intra-individual variation of the association between mercury levels and liver dysfunction. Therefore, panel study, detecting within-person changes across time and increasing statistical power by repeatedly measuring the levels of mercury and liver enzymes, was better than single-measured cross-sectional study. A recent study reported that elderly adults, particularly those aged 60–69 years and Asians residing in the US communities had the highest exposure prevalence of methylmercury according to the National Health and Nutrition Examination Survey (NHANES) 2011–2012 [19]. In the Korea National Health and Nutrition Examination Survey (KNHANES, 2008–2012), mean values of blood mercury concentration were 5.07 ± 0.07 μg/L in males and 3.59 ± 0.04 μg/L in females [20] and they were higher than NHANES. Therefore, there is a need to investigate the elderly Asians vulnerable to mercury exposure for the association with liver function.

The objective of this study was to determine the association between blood mercury concentrations and liver enzyme levels (AST, ALT, and GGT) using repeated measurements in an elderly population. In addition, we investigated the interaction of alcohol consumption and blood mercury concentrations on liver enzyme levels. We also assessed the mediation effect of oxidative stress on the relationship between mercury exposure and liver function because some studies suggested that oxidative stress mediated mercury-induced hepatotoxicity in rats [21].

## Methods

### Study population

We recruited 560 participants who regularly visited a community welfare center located in Seongbuk-gu, Seoul, Korea. The inclusion criteria were an age of 60 years or older, the ability to communicate with interviewers and volunteered to participate in the study. Fasting blood was collected up to five times (from August to December 2008, from April to October 2009, from March to August 2010, from October 2012 to June 2013, and from October 2013 to June 2014) from each participant. Recruitment period was from 2008 to 2010 and follow-up period was from 2009 to 2014. Additional file 1: Figure S1 showed a detail of recuitment and follow-up. Information on demographic characteristics, health behavior and medical history and a food frequency questionnaire to assess dietary intake for the past year was obtained at the time of enrollment. Our study design was a panel study that we collected repeated measures (blood mercury and liver enzymes) from the same sample at every visit.

We excluded participants who had chronic hepatitis, acute hepatic disease, fatty liver and liver cancer and those who lacked information for blood mercury concentrations and liver enzyme levels. A total of 550 individuals and 1222 observations were included in the analysis. Among the 550 participants, 173, 177, 140, 25 and 35 individuals had blood samples collected once, twice, three times, four times and five times, respectively.

The study protocol was approved by the institutional review board at Seoul National University Hospital, Seoul, Republic of Korea (IRB no. C-1209-006-424), and each study participant provided written informed consent. This study was performed in accordance with the Declaration of Helsinki.

### Measurement of blood mercury

To measure mercury concentrations in blood, 1 ml of whole blood was drawn from each participant and then sealed in a heparin containing tube (EDTA tube by BD REF 367856). The blood samples were stored at − 70 °C until analysis. The sample was initially dried in an oxygen stream passed through a quartz tube located inside a controlled heating coil. The combustion gases were further decomposed on a catalytic column at 750 °C. Mercury vapor was collected on a gold amalgamation trap and then desorbed for quantification. Blood mercury concentration was determined by flow injection cold-vapor atomic absorption spectrometry (AAS) (DMA-80, Milestone, Bergamo, Italy). The laboratory analyses were performed using standardized quality-control procedures. An internal control was used for each series of analyses. The validity of the mercury level measurement was verified by periodically participating in an external quality control program (the German external quality assessment scheme (G-EQUAS) and the CDC’s Lead and Multi-element Proficiency (LAMP) program). The limit of detection (LOD) was 0.158 μg/L, and no sample had a mercury level below the LOD.

### Obesity and lifestyle measurement

Trained health technicians measured body weight (kg) and height (cm) following standardized procedures for all participants. Body mass index (BMI) was calculated as body weight in kilograms divided by height in meters squared. Overweight was defined as 25 kg/m^2^ or higher using the World Health Organization (WHO) criteria [22]. Study participants were also split into three groups by smoking status: less than 20 packs in lifetime smoking (non-smoker), greater than 20 packs in lifetime and don’t smoke now (previous smoker), and greater than 20 packs in lifetime and smoke now (current smoker). We used only two categories for alcohol consumption: “regular drinkers and non-drinkers”.

### Liver enzyme measurement

Blood samples (up to 3 mL) were collected from each participant at every visit using a BD vacutainer (Becton Dickinson, Franklin Lakes, New Jersey, USA) containing K_2_EDTA (Becton Dickinson) and preserved at −70 °C. Serum AST, ALT and GGT were subsequently analyzed by an autobiochemical analyzer (Hitachi 7600-II, Hitachi High-Technologies Co., Tokyo, Japan). Pureauto S AST, Pureauto S ALT, and Pureauto S GGT (Daiichi Pure Chemicals, Tokyo, Japan) were used as reagents.

### Measurement of malondialdehyde

We measured urinary levels of malondialdehyde (MDA) as an oxidative stress biomarker. Urinary MDA levels were determined by measuring thiobarbituric acid reactive substances as follows: 50 μl of urine was mixed with 300 μl of 0.5 M phosphoric acid solution and 150 μl of 23 mM TBA solution (Sigma-Aldrich T-5500, Steinheim, Germany) and heated at 95 °C for 1 h. After cooling on ice, the mixture was vortexed with 500 μl of methanol and centrifuged at 5000 × *g*. Absorbance of the supernatant was measured at 532 nm using HPLC–UV. The mobile phase was potassium phosphate (0.05 mol/L; pH 6.8) and methanol (58:42, v/v).

### Statistical analysis

Because the concentrations of the three liver enzymes were not normally distributed, log-transformed values were used in the analysis. The geometric means (GMs) (95% confidence interval (CI)) of blood mercury were calculated by demographic characteristics. Blood mercury levels were divided into quartiles. Abnormal liver function tests were defined as serum AST >34 U/L in men and >40 U/L in women; serum ALT >35U/L in both men and women; and serum GGT >48 U/L in men and >29 U/L in women [23].

We performed a multivariate analysis using a generalized estimating equations (GEE) model with exchangeable correlation structure to estimate the effects of blood mercury on liver function taking account for correlated structure of data due to repeated measures over time. We modeled log-transformed liver enzyme levels as a normal distribution and abnormal liver function as a binomial distribution.

We adjusted potential confounder which were considered as a confounders in previous epidemiologic study [17] (age [years], sex [male and female], overweight [BMI ≥ 25 kg/m^2^], alcohol consumption [regular drinkers or non-drinkers], smoking [nonsmoker, previous smoker, current smoker], education [≤elementary school graduate, ≥middle school graduate]) or variables which was associated with nonalcoholic fatty liver disease (high-density lipoproteins (HDL) cholesterol [mg/dL], regular exercise [yes or no] and total calorie intake [kcal]) [24, 25].

Alcohol intake is a important risk factor for liver disease [26], if there is an interaction between alcohol and mercury for liver function, people with high mercury levels should limit alcohol intake even more. To evaluate the interaction effects of alcohol consumption in the association between mercury concentrations and liver function, we used median for mercury cut-off concentration (men = 3.77 μg/L; women = 2.59 μg/L). We stratified data into four groups (non-drinkers and low mercury level, non-drinkers and high mercury level, regular drinkers and low mercury level, and regular drinkers and high mercury level), and estimated odds ratio (OR) of having abnormal liver function compared to the reference group (non-drinkers and low mercury level).

To test the mediation effect of oxidative stress on the association between mercury concentrations and liver enzyme levels, we used multiple linear regression models and mediaiton analysis that was developed in SAS macros by Jasti et al. [27] based on the formula of Mackinnon and Dwyer [28]. After assessing the linear relationship of blood mercury levels with oxidative stress and liver function, a Sobel test [29] was conducted for testing the significance of a mediation effect. Mercury levels were used as exposure variables and MDA for oxidative stress was used as mediators. Log-transformed liver enzyme levels were used as the outcome variables. The mediation analysis was conducted on all participants, then non-drinkers and regular drinkers, separately.

To visualize a linear relationship, we used generalized additive mixed models of R (package ‘gamm4’) with cubic smoothing spline (*k* = 3).

All of the analyses applied two-sided tests, and we considered a *p*-value lower than 0.05 to be significant. Statistical analyses were conducted using R version 2.15.2 (The Comprehensive R Archive Network: https://cran.r-project.org/) and SAS software version 9.3 (SAS Institute Inc., Cary, NC, USA).

## Results

Participants’ characteristics and geometric mean (95% CI) mercury levels at the first visit are shown in Table 1. The mean age of 550 participants was 70.7 years (range, 60–87 years), and the ratio of men to women was approximately one to three. Men, ages 60–69 years, higher education attainment, overweight, regular alcohol drinkers, and higher calorie intake showed higher total blood mercury. Participants who had higher blood mercury levels were significantly higher in abnormal AST and ALT (*p* < 0.05).

**Table 1:** Geometric mean and 95% confidence intervals of blood mercury levels by participant characteristics at the first visit

Characteristic	No.	GM (95% CIs)	*p*-value
Total	550	2.78 (2.65, 2.92)	
Age (years)			
60–69	246	3.31 (3.10, 3.54)	<.0001
70–79	281	2.70 (2.55, 2.87)	
80-	23	1.99 (1.49, 2.64)	
Sex			
Men	141	3.67 (3.36, 4.00)	<.0001
Women	409	2.70 (2.57, 2.84)	
Education			
≤ Elemental school graduate	303	2.68 (2.52, 2.84)	<.0001
≥ Middle school graduate	231	3.28 (3.07, 3.51)	
Missing	16		
BMI (kg/m^2^)			
< 25	308	2.79 (2.62, 2.98)	0.015
≥ 25	242	3.09 (2.91, 3.29)	
Cigarette smoking			
Non smoker	472	2.86 (2.73, 3.01)	0.077
Previous smoker	34	3.41 (2.88, 4.03)	
Current smoker	29	3.32 (2.81, 3.93)	
Missing	15		
Alcohol			
Non-drinker	414	2.78 (2.64, 2.92)	<.0001
Regular drinker	119	3.48 (3.14, 3.87)	
Missing	17		
AST (U/L)			
Men:≤34; Women:≤40	532	2.90 (2.77, 3.03)	0.007
Men:>34; Women:>40	18	3.74 (3.15, 4.45)	
ALT (U/L)			
≤ 35	513	2.87 (2.74, 3.01)	0.003
> 35	37	3.75 (3.23, 4.36)	
GGT (U/L)			
Men:≤48; Women:≤29	461	2.87 (2.74, 3.02)	0.097
Men:>48; Women:>29	89	3.18 (2.84, 3.57)	
HDL-Cholesterol (mg/dL)			
Men:<40; Women < 50	328	2.97 (2.81, 3.14)	0.344
Men:≥40; Women ≥ 50	222	2.85 (2.64, 3.07)	
Energy intake (Kcal)			
< 2000	262	2.76 (2.60, 2.94)	0.014
> 2000	188	3.13 (2.89, 3.40)	
Missing	100		

Student’s *t*-test and ANOVA (analysis of variance) used to compare means across categories

*Abbreviations*: *ALT* Alanine transaminase, *AST* aspartate aminotransferase, *BMI* body mass index, *CIs* confidence intervals, *GGT* Gamma-glutamyl transferase; GM, geometric mean

The arithmetic and geometric mean, minimum, maximum, quartiles of blood mercury at each visit are shown in Additional file 1: Table S1. The arithmetic and geometric mean of mercury in all samples were 3.29 and 2.81 μg/L, respectively.

When adjusted by sex, age, smoking status, drinking status, exercise, education, HDL cholesterol, overweight, and calorie intake using the GEE model, those with the 4th quartile of blood mercury showed significantly higher AST and ALT compared to those with 1^st^ quartile of blood merury, and those with the 3^th^ or 4^th^ quartiles of blood mercury had significantly higher GGT compared to those with the first quartile (Table 2).

**Table 2:** Estimated association of log transformed liver enzyme and blood total mercury using GEE model

Mercury	AST		ALT		GGT	
	Estimate	*p*-value	Estimate	*p*-value	Estimate	*p*-value
Q1	Ref		Ref		Ref	
Q2	0.01 (0.02)	0.733	0.04 (0.03)	0.204	0.04 (0.03)	0.201
Q3	0.03 (0.03)	0.228	0.04 (0.03)	0.184	0.08 (0.04)	0.044
Q4	0.07 (0.03)	0.027	0.12 (0.04)	0.002	0.12 (0.04)	0.008

Covariates: sex, age, smoking status, drinking status, exercise, education, high-density lipoproteins cholesterol, overweight and calorie

*Abbreviations*: *ALT* Alanine transaminase, *AST* aspartate aminotransferase, *GEE* generalized estimating equations, *GGT* Gamma-glutamyl transferase, *Q* quartile: 1^st^ Q (<2.50 for men, <1.86 for women), 2^nd^ Q (2.50 ≤ and <3.77 for men, 1.86 ≤ and <2.59 for women), 3^rd^ Q (3.77 ≤ and <5.41 for men, 2.59 ≤ and <3.53 for women), 4^th^ Q (≥5.41 for men, ≥3.53 for women)

Results observed using dichotomized outcomes for serum liver enzymes (normal vs. abnormal) are shown in Table 3. Among individuals who had the highest quartile of total blood mercury levels, the OR of having abnormal ALT level was 3.10 (95% CI 1.17–8.24) compared to those with the lowest quartile.

**Table 3:** Odds ratios of liver function tests to total blood mercury using GEE model

Mercury	AST		ALT		GGT	
	OR	95% CI	OR	95% CI	OR	95% CI
Abnormal liver test						
Q1	Ref		Ref		Ref	
Q2	1.30	0.41–4.11	2.01	0.83–4.90	1.34	0.86–2.07
Q3	1.68	0.49–5.76	2.05	0.77–5.43	1.02	0.62–1.67
Q4	2.70	0.95–7.68	3.10	1.17–8.24	1.29	0.79–2.13

Covariates: sex, age, smoking status, drinking status, exercise, education, high-density lipoproteins cholesterol, overweight, calorie

*Abbreviations*: *ALT* Alanine transaminase, *AST* aspartate aminotransferase, *CI* confidence interval, *GEE* generalized estimating equations, *GGT* Gamma-glutamyl transferase, *Q* quartile: 1^st^ Q (<2.50 for men, <1.86 for women), 2^nd^ Q (2.50 ≤ and <3.77 for men, 1.86 ≤ and <2.59 for women), 3^rd^ Q (3.77 ≤ and <5.41 for men, 2.59 ≤ and <3.53 for women), 4^th^ Q (≥5.41 for men, ≥3.53 for women), *OR* odds ratio

**Fig. 1:**
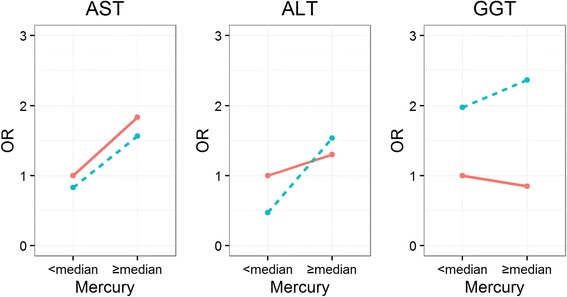
Odds ratios for abnormal AST, ALT, and GGT by blood mercury using generalized estimating equations model. Abbreviations: ALT, Alanine transaminase; AST, aspartate aminotransferase; GGT, Gamma-glutamyl transferase; Q, quartile; OR, odds ratio

The interactive effect of blood mercury level in the relationship between alcohol consumption and abnormal liver function (AST, ALT and GGT) is shown in Fig. 1. The group with high mercury level and regular alcohol consumption habit showed significantly high OR of having abnormal GGT (OR 2.37, 95% CI 1.19–4.69) compared to the reference group (non-drinkers and those with low mercury level) with an interaction *P*-value < 0.001.

The measures of mediation for MDA in the relationship between blood mercury levels and liver enzymes in all participants and the non-drinker group are presented in Table 4. For both all participants and non-drinking group, MDA was not significantly mediated.

**Table 4:** Tests and measures of mediation for MDA in the relationship between mercury and liver function

Mediation variable	Outcome	Indirect effect	S.E	*P* value for Sobel test	Percent (%) of the total effect that is mediated
All participants					
MDA	AST	1.0× 10^−5^	6.3× 10^−5^	0.872	11.92
	ALT	−1.2× 10^−5^	7.3× 10^−5^	0.873	12.17
	GGT	1.0× 10^−5^	6.6× 10^−5^	0.879	19.38
Non-drinkers					
MDA	AST	1.1× 10^−4^	3.2× 10^−4^	0.735	3.24
	ALT	−2.2× 10^−4^	4.1× 10^−4^	0.583	7.37
	GGT	1.2× 10^−5^	3.3 × 10^−4^	0.971	27.29

*Abbreviations*: *ALT* Alanine transaminase, *AST* aspartate aminotransferase, *BMI* body mass index, *CI* confidence interval, *GGT* Gamma-glutamyl transferase, *MDA* Malondialdehyde, *S.E* standard error

We showed linear relationship between blood mercury and liver function using generalized additive mixed model in Additional file 1: Figure S2.

## Discussion

To our knowledge, this was the first study using repeated measurements which considered within-person change of mercury exposure and liver function across time. The findings of the present study suggest that total blood mercury levels were associated with elevated liver enzyme concentration in the elderly. We also found an interaction effect of alcohol consumption and mercury concentration on liver function. Fatty liver changes accompanied by increased BMI may contribute partly to liver function impairment in the association with mercury exposure within non-drinkers. A mediation analysis was performed to demonstrate the mechanism of mercury’s effect on liver function. In our study, oxidative stress, which was suggested from another study in rat [21, 30], was not found to mediate the interaction between mercury and liver function.

The geometric mean of total blood mercury levels in the present study was 2.81 μg/L (2.73, 2.89), which was relatively higher than the levels from other countries’ surveys. The geometric mean for total blood mercury was 0.23 μg/L in the German Environmental Survey IV (2003–2008) [31]. In the study based on the 2011–2012 NHANES, the geometric mean of total blood mercury level was 0.70 (0.62–0.80) μg/L [men 0.71 (0.62–0.81) μg/L, women 0.69 (0.61–0.79) μg/L], and the Asian group had the highest blood mercury level [1.86 μg/L (1.58, 2.19)] [19] among the US population. Our study participants had lower blood mercury concentration, compared to KNHANES (2008–2012) [men 5.07 (5.00–5.14) μg/L and women 3.59 (3.55–3.63) μg/L] [20].

The present findings also supported some previous epidemiologic studies. Cave et al. [16] showed that whole blood mercury level was associated with ALT elevation in NHANES 2003–2004. Those authors suggested that the organic form (methylmercury) of mercury was associated with liver disease. Lee et al. [17] reported that the OR of having serum AST and ALT levels higher than the median among subjects in the highest quartile of blood mercury were 1.53 (1.26–1.85) and 1.95 (1.63–2.33) compared to the lowest quartile, respectively, using the 2008–2012 KNHANES. Seo et al. [18] demonstrated that the adjusted OR (95% CI) for high GGT in the highest quartile compared to the lowest quartile of blood mercury levels was 2.59 (1.51–4.43) in men and 2.03 (1.13–3.67) in women using the 2010 KNHANES.

Although the mechanism of mercury-induced hepatotoxicity has not been clarified, some studies suggested that mercury produces reactive oxygen species and depresses antioxidant system components, and the resulting oxidative stress triggers hepatotoxicity [12, 13, 32]. In the present study, when we performed a mediation analysis for oxidative stress biomarkers, we could not find significant results in all of the participants or the non-drinker group.

Alcohol consumption is a major risk factor for liver disease, and one-third of liver cirrhosis cases are attributable to heavy alcohol consumption [26]. However, there was still a significant mercury effect on liver function when alcohol consumption was adjusted in the multivariate model. We also observed a significant association between the highest quartile of mercury levels and ALT in the non-drinker group (Additional file 1: Table S2). In addition, we found the interaction between alcohol consumption and mercury exposure for liver function. WHO reported that alcohol per capita consumption in Korea was 12.3 l, which is higher than 6.2 l in world [33]. Mercury level in Koreans is also higher than most other countries [34]. Therefore, liver dysfunction could be more prevalent among Koreans, particularly with alcohol drinking and high mercury level.

Our results showed that blood mercury concentrations are most associated with ALT among the three liver enzymes. Both AST and ALT are released from damaged hepatocytes into the blood, but AST is also released from several non-hepatic tissues including the heart, skeletal muscle and blood. ALT is more specific to hepatocellular liver injury and disease [35]. GGT is the enzyme that catalyzes the hydrolysis of glutathione and transfer of the γ-glutamyl group from one peptide to another or to an amino acid in the liver [36]. Because the blood level of GGT is a more sensitive biomarker for alcohol consumption [37] than ALT and AST, GGT was appropriate for revealing the synergistic effect between alcohol and mercury on liver function.

The present study has some limitations. First, this study was conducted in an elderly population; thus, the results cannot be generalized to general population. Also, we recruited participants at only one center in Seoul, didn’t randomly select the participants or didn’t use systematized sampling procedures. So this data is not representative sample in relation to the South Korean elderly population. Second, although we used repeated analysis, we could not exclude the possibility of reverse causation. Lin et al. [38] found that MeHg represents a higher fraction of the total circulating Hg among those with elevated all three liver enzyme (AST, ALT and GGT) using NHANES 2003–2008. They explained that compromised liver function may result in decreased demethylation and therefore an increased organic fraction (MeHg) of blood total Hg. Third, we measured only total Hg in blood and we did not measure organic mercury. Mortensen et al. [19] showed that the NHANES 2011–2012 provided data to examine total, inorganic, methyl, and ethyl mercury directly in whole blood. And they found that total Hg and MeHg concentrations were highly correlated, and the ratio of MeHg to THg was higher for Asians (0.85). So blood tests are generally used to measure the organic mercury because of the high rate of uptake of methylmercury in red blood cells [39]. Fourth, although we excluded participants with chronic hepatitis, acute hepatic disease, fatty liver and liver cancer, it is possible that we may have included participants who had mild liver dysfunction caused by the other factors than mercury exposure. Fifth, information on fish intake was not available, therefore, it couldn’t be adjusted in analysis. Sixth, in mediation analysis, SAS macros by Jasti et al. based on the formula of Mackinnon and Dwyer did not provide correlation structure, so all variable was considered independent. Finally, we used only two categories for alcohol consumption: “regular drinkers and non-drinkers”. We couldn’t calculate alcohol consumption per week so we couldn’t classify into heavy drinker or moderate drinker.

## Conclusions

We conducted repeated measurements of total blood mercury to evaluate the effect of environmental exposure to mercury on liver function in an elderly population. Our findings suggest that exposure to mercury increase the risk of liver function abnormality and interact with alcohol consumption for elevating the risk.

## Additional file

Additional file 1: Table S1. Distribution of mercury at each visit and overall study. **Table S2.** Estimated association between log transformed liver enzymes and total blood mercury levels using generalized estimating equations model in non-drinkers. **Figure S1.** Flow chart for study participants. **Figure S2.** Generalized additive mixed model for total mercury concentrations and three liver enzymes. Additionally adjusted for sex, age, smoking status, drinking status, exercise, education, high-density lipoproteins cholesterol, overweight and calorie. (PDF 147 kb)

## Abbreviations

AAS: Atomic absorption spectrometry
ALT: Alanine aminotransferase
AST: Aspartate aminotransferase
BMI: Body mass index
CI: Confidence interval
GEE: Generalized estimating equations
G-EQUAS: The German external quality assessment scheme
GGT: Gamma glutamyl transferase
GMs: Geometric means
HDL: High-density lipoproteins
KNHANES: Korean National Health and Nutritional Examination Survey
LAMP: Multi-element Proficiency
LOD: Limit of detection
MDA: Malondialdehyde
NHANES: National Health and Nutrition Examination Survey
OR: Odds ratio
