# Supplementary material for: Blood mercury concentrations are associated with decline in liver function in an elderly population: a panel study
Source: Environ Health. 2017 Mar 4;16:17. doi: 10.1186/s12940-017-0228-2 (PMC5336614; doi:10.1186/s12940-017-0228-2)
Supplement: Additional file 1: Table S1. — Distribution of mercury at each visit and overall study. Table S2. Estimated association between log transformed liver enzymes and total blood mercury levels using generalized estimating equations model in non-drinkers. Figure S1. Flow chart for study participants. Figure S2. Generalized additive mixed model for total mercury concentrations and three liver enzymes. Additionally adjusted for sex, age, smoking status, drinking status, exercise, education, high-density lipoproteins cholesterol, overweight and calorie. (PDF 147 kb) [file 12940_2017_228_MOESM1_ESM.pdf]

Table S1. Distribution of mercury at each visit and overall study

	visit	N	AM	GM	Percentile				
					0(Min)	25th	50th	75th	100(Max)
Mercury	First	550	3.38	2.78	0.605	1.852	2.638	3.979	71.001
	Second	377	3.23	2.82	0.588	2.032	2.805	3.812	12.960
	Third	200	3.28	2.87	0.615	2.012	2.960	4.019	20.151
	Fourth	60	3.16	2.86	0.843	2.059	2.965	3.851	7.246
	Fifth	35	2.89	2.65	0.964	2.023	2.661	3.658	6.748
	Total	1222	3.29	2.81	0.588	1.955	2.768	3.931	71.001

Abbreviations: AM, Arithmetic mean; GM, Geometric mean; Max, maximum; Min, minimum

Table S2. Estimated association between log transformed liver enzymes and total blood mercury levels using generalized estimating equations model in non-drinkers

	AST		ALT		GGT	
Mercury	Estimate	p-value	Estimate	p-value	Estimate	p-value
Non-drinker						
Q1	Ref		Ref		Ref	
Q2	0.01(0.03)	0.705	0.03(0.03)	0.374	0.01(0.04)	0.764
Q3	0.03(0.03)	0.376	0.02(0.04)	0.556	0.04(0.04)	0.399
Q4	0.06(0.03)	0.099	0.10(0.05)	**0.022**	0.06(0.04)	0.215

Covariates: sex, age, smoking status, drinking status, exercise, education, high-density lipoproteins cholesterol, overweight and calorie

Abbreviations: ALT, Alanine transaminase; AST, aspartate aminotransferase; GGT, Gamma-glutamyl transferase; Q, quartile

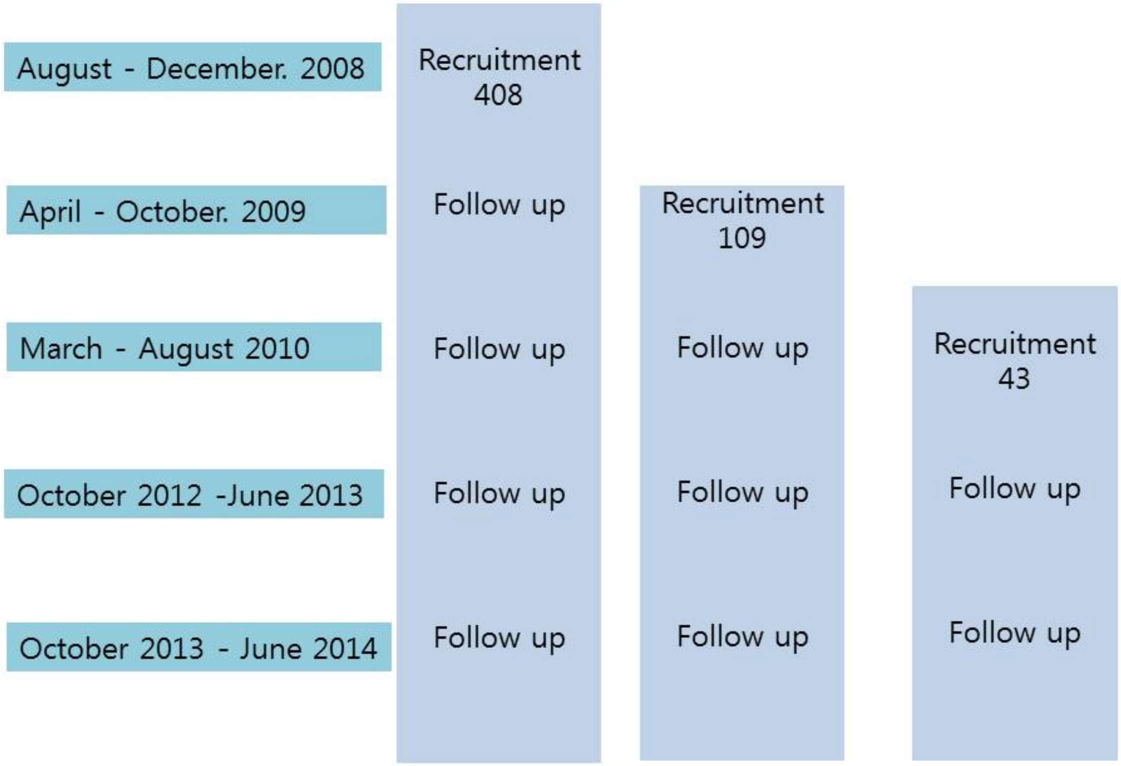

Fig S1. Flow chart for study participants

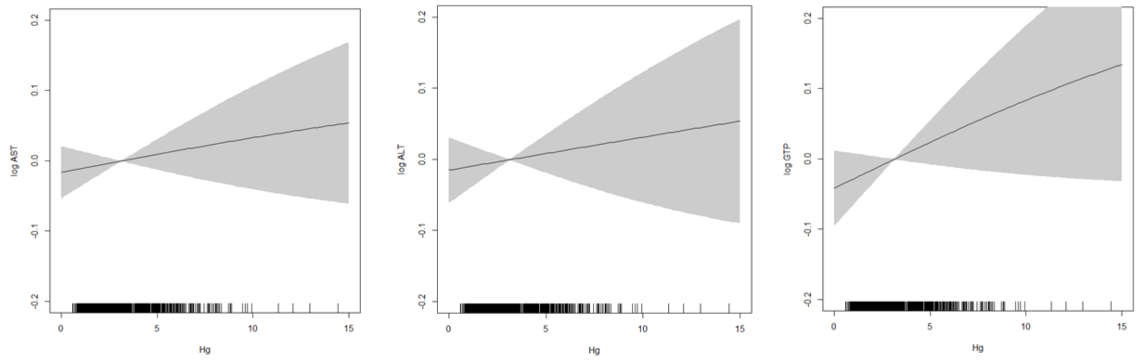

Fig S2. Generalized additive mixed model for total mercury concentrations and three liver enzymes. Additionally adjusted for sex, age, smoking status, drinking status, exercise, education, high-density lipoproteins cholesterol, overweight and calorie
